# A Note on the Risk of Infections Invading Unaffected Regions

**DOI:** 10.1155/2018/6289681

**Published:** 2018-07-04

**Authors:** Marcos Amaku, Francisco Antonio Bezerra Coutinho, Margaret Armstrong, Eduardo Massad

**Affiliations:** ^1^School of Medicine, University of Sao Paulo, Sao Paulo, Brazil; ^2^School of Applied Mathematics, Fundação Getúlio Vargas, Rio de Janeiro, Brazil; ^3^College of Natural and Life Sciences, The University of Derby, Derby, UK; ^4^London School of Hygiene and Tropical Medicine, London, UK

## Abstract

We present two probabilistic models to estimate the risk of introducing infectious diseases into previously unaffected countries/regions by infective travellers. We analyse two distinct situations, one dealing with a directly transmitted infection (measles in Italy in 2017) and one dealing with a vector-borne infection (Zika virus in Rio de Janeiro, which may happen in the future). To calculate the risk in the first scenario, we used a simple, nonhomogeneous birth process. The second model proposed in this paper provides a way to calculate the probability that local mosquitoes become infected by the arrival of a single infective traveller during his/her infectiousness period. The result of the risk of measles invasion of Italy was of 93% and the result of the risk of Zika virus invasion of Rio de Janeiro was of 22%.

## 1. Introduction

Many countries where infectious diseases had been considered controlled in past decades are reporting the invasion of some exotic and frequently unknown infectious diseases that are spread by infected travellers/immigrants [1, 2]. Historical examples of disease invasion are numerous. A particular tragic invasion was the invasion of Europe by the Black Death in XIV century, which started probably in China and travelled by ship until reaching European shores where it decimated from a quarter to a half of the European population [3, 4]. Another tragic invasion occurred in the beginning of the last century when between 50 and 100 million individuals worldwide died victims of the Spanish Flu, which probably started in an American army barrack in the United States in 1918 and rapidly was spread by travellers to others areas of the world [5, 6]. Many years later SARS affected many countries, spread by infected travellers [7, 8]. In 2009 the swine flu pandemic (H1N1) frightened the world, exemplifying the dangers of international spread of communicable diseases [9, 10]. The recent outbreak of Ebola, which spilled over to the United States showed how individuals travelling from infected to uninfected areas of the world can be dangerous [11, 12]. Other recent examples of infectious diseases spreading by travellers include the Zika virus outbreak in Latin America, in particular in Brazil, which was imported from the French Polynesia by infected travellers [13]. Now, Europe is in the grip of a huge measles outbreak caused by the recent waves of nonvaccinated immigrants and the low vaccination coverage of some European countries [14–17].

Human mobility networks are playing an increasing role in the spread of communicable diseases [1, 19], at both the international and the national levels and even between different districts in the same city. Infected travellers can introduce infectious agents to new areas and populations [3]. The highest risk for global health now is the indisputable fact that a traveller with an infectious disease can reach virtually any part of the world within 24 h. The current staggering volume, speed, and reach of travel are unprecedented, showing a virtually uninterrupted growth—from 25 million in 1950 to 278 million in 1980, 528 million in 1995, and 1087 million in 2013 [20]. Asia and the Pacific recorded the fastest relative growth across all World Tourism Organization (UNWTO) regions, with a 6% increase in international arrivals per year in recent years and Asia is the epicentre of many infectious diseases [20]. Africa, another continent with many emerging infectious diseases, saw an increase of 5%. International tourist arrivals worldwide are expected to increase by 3.3% per year from 2010 to 2030 to reach 1.8 billion by 2030, according to UNWTO's long-term forecast Tourism Towards 2030 [20].

Current methodological approaches to estimate the risk of diseases in travellers still have many shortcomings [9, 21–23]. For example, estimations based on notifications of imported cases underestimate the risk. This is so because many diseases are either not notifiable to authorities or even if legally notifiable are underreported, as not every traveller will report her/his condition to healthcare providers. Moreover, many imported diseases may go unnoticed because of a high frequency of asymptomatic cases that may also contribute to the transmission of diseases. In the absence of good data on importation and exportation of infectious diseases via international travellers, mathematical models can provide an additional tool for the estimating the risks involved.

Situations where a relatively small number of infected individuals travel to previously disease-free areas are particularly critical. In these cases, the deterministic formulation of risk is not sufficient and hence the necessity of new probabilistic models to estimate the risk of disease invasion. Here, a probabilistic model is developed, taking into account air travel volume, force of infection in the country of disembarkation, and herd immunity due to either background immunity or immunization coverage by vaccination. Two distinct situations related to the spread of infections by travellers are considered, namely, a directly transmitted infection and a vector-borne infection. We used the case of measles in Europe and the potential spread of Zika virus to disease-free but Aedes-infected areas (Rio de Janeiro, Brazil) as a proof of principle and to illustrate the methods. Uncertainty analysis was considered as confidence intervals and the simulation were carried out by solving the specific equations analytically.

## 2. Case 1. Directly Transmitted Infection

The model assumes that a density of infected individual, *I*_*H*_^*T*^(0) (see [24]), arrived at *t* = *t*_0_ and remains infective for a period of (*μ*_*H*_ + *γ*_*H*_ + *α*_*H*_)^−1^weeks, that is,

(1) IHTt=IHT0exp⁡−μH+γH+αHt−t0θt−t0

where *μ*_*H*_, *γ*_*H*_, and *α*_*H*_ are the natural mortality rate, the recovery rate from infection, and the disease-induced mortality rate, respectively, and *θ*(*t* − *t*_0_) is the Heaviside step function. If the region where these infected travellers arrive had an area *A* the number of them is *I*_*H*_^*T*^(0)*A*.

The total number of measles cases, *Measles*_*cases*_, infected by these travellers, one year after its introduction (Δ = 52 weeks), is given by

(2) $$\begin{array}{c} Measles_{cases}=\overset{t_{0}+\Delta }{\underset{t_{0}}{\int }}\sigma I_{H}^{T}\left(\;t\;\right)\frac{S_{H}}{N_{H}}dt \end{array}$$

where *σ* is the potentially infective contact per unit time between infected and susceptible individuals and *S*_*H*_ and *N*_*H*_ are the susceptible and total population, respectively.

The risk of measles invasion of a previously unaffected country, *Risk*_*measles*_, can be defined as the probability that at least one autochthonous case be produced by the arrival of one single infected individual at the area during his/her infectiousness period. To calculate this risk, we assumed a nonhomogeneous simple birth process [25], which describes the introduction of the disease. Note that we are interested only in the estimation of the risk of infection and, therefore, recovery from the infection is not considered in the risk estimation.

Let *P*_*n*_(*t*) be probability of *n* cases. The probability generating function of such process is *P*(*x*, *t*) = ∑_*n*_*P*_*n*_(*t*)*x*^*n*^. After some calculation [25] we obtain

(3) $$\begin{array}{c} P\left(\;x,t\;\right)={\left\{\;1+\frac{1}{e^{\rho \left(t\right)}/\left(x-1\right)-\int_{0}^{t}\lambda \left(\tau \right)e^{\rho \left(\tau \right)}d\tau }\;\right\}}^{a} \end{array}$$

where *x* is a dummy variable, *a* = *I*_*H*_(0)*A*, and *λ*(*t*) is the “birth rate of the process”; in this case the incidence of the infection and is defined as *λ*(*t*) = *σ*(*I*_*H*_^*T*^(*t*)*S*_*H*_(*t*)/*N*_*H*_), where *S*_*H*_(*t*) is obtained by solving (A.1) from the Appendix using the parameters obtained in the next section and described in Table 2. In (3), *ρ*(*t*) = −∫_0_^*t*^*λ*(*τ*)*θ*(*τ* − *t*_0_)*dτ*, *τ* is a dummy variable and *θ*(*τ* − *t*_0_) is the Heaviside step function, introduced to simulate the moment the infected traveller arrives. Note that *ρ*(*t*) has no physical meaning and was introduced to simplify the notation.

We assumed that the region to be studied has an area *A* and that the number of infected travellers arriving at *t* = *t*_0_ is *a* = *I*_*H*_(0)*A*. We set *a* = 1 from now on.

Expanding (3) in powers of *x* we find that the risk, that is, the probability of having *n* infected individual at time *t*, denoted *Risk*_*measles*_(*n*, *t*) as

(4a) Riskmeaslesn,t=∑j=0min⁡n,aaja+n−j−1a−1αta−jβtn−j·1−αt−βtj

The risk (probability) of having no infected individuals is

(4b) $$\begin{array}{c} Risk_{measles}\left(\;0,t\;\right)=\alpha {\left(\;t\;\right)}^{a} \end{array}$$

In (4a) and (4b)

(5) αt=1−1eρt+∫0tλτθτ−t0eρτdτ,βt=1−eρteρt+∫0tλτθτ−t0eρτdτ.

Note that neither *α*(*t*) nor *β*(*t*) have any physical meaning and were introduced to simplify the notation. Note also that both *α*(*t*) and *β*(*t*) increase with time reaching a constant value denoted *α* and *β*, respectively.

The probability of at least 1 autochthonous case in a previously unaffected region can be calculated as follows:

(6) Riskmeasles=PNumber  of  secondary  cases>1=1−αa

The upper and lower values of 95% confidence intervals for the number of secondary cases are given by

(7) mlower=ln⁡0.975+ln⁡1−αln⁡βmupper=ln⁡0.025+ln⁡1−αln⁡β

### 2.1. The Case of Measles in Europe

In 2017, Europe reported more than 21,000 cases of measles, including 35 deaths (a mortality rate of 6.0×10^−5^ month^−1^)[14]. This represents a fourfold increase from 2016 when just 5,273 cases were reported. This outbreak was due to two factors: first, there was an overall decline in routine immunization coverage, with particularly low coverage among marginalised groups, in addition to interruption in vaccine supply or underperforming disease surveillance; second, large numbers of nonvaccinated immigrants flooded Europe in previous years (5,136,383 since 2011, according to the European Asylum Support Office, [17]).

According to the Communicable Disease Threat Report [14], the highest numbers of cases of measles since 1 January 2017 were reported in Romania (8,274), Italy (4,885), and Germany (919), the three countries which received the highest numbers of immigrants from undervaccinated areas [17].

Let us analyse the case of Italy where, according to the ECDC [14], the measles vaccination coverage is less than 84% (well below the herd immunity threshold of 95%). If we assume for 2017 the same proportion of immigrants from measles undervaccinated countries to Europe as the one observed in 2015 (3% see [17]), then of the around 700 thousand immigrants that arrived in Europe in 2017, around 21 thousand settled in Italy. In that year, Italy reported 4,885 measles cases, as mentioned above. Assuming a vaccination coverage of 80%, it is possible to estimate the risk of measles introduction by considering the effective reproduction number *R*(*t*) = *R*_0_(*S*_*H*_(*t*)/*N*_*H*_), where *R*_0_ = *σ*/(*μ*_*H*_ + *γ*_*H*_ + *α*_*H*_) is the basic reproduction number [26]. So, the effective reproduction number reaches a value of 5, a value compatible with the 20% susceptible individuals, the proportion of nonvaccinated individuals in Italy. This is because the basic reproduction number of measles is around 25 and therefore *R*(*t*) = *R*_0_(*S*_*H*_(*t*)/*N*_*H*_) = 25*∗*0.2 = 5. Assuming a recovery rate *γ*_*H*_ = 3.33 month^−1^, a natural mortality rate *μ*_*H*_ of 1.10×10^−3^ month^−1^, a disease-induced mortality of *α*_*H*_ = 6.0×10^−5^ month^−1^, and using the expression for *R*_0_, we estimated that the potentially infective contact rate is *σ* = 10 month^−1^. By using these parameters it is possible to calculate *λ*(*t*) = *σ*(*I*_*H*_^*T*^(*t*)*S*_*H*_(*t*)/*N*_*H*_), and *α* of (6). From this equation it follows that the probability that one single infective immigrant, arriving in Italy on January 1^st^, could generate at least one secondary, autochthonous measles case with a probability of around 93%, which is reasonably high risk. The 95% CI for the number secondary cases is (0.89 - 1.40). In other words, one secondary case will be produced by a single traveller with confidence interval between 0.89 and 1.40 cases per unit area.

As we estimated the basic reproduction number of measles in Italy at the time of the outbreak as equal to 5, it would be interesting to estimate the number of infected individuals necessary to arrive to guarantee that at least one secondary infection would be generated. In other words, what would be the value of *a* in (4a) that would make [1 − *p*(0)]≅1. The result is that seven infected immigrants would guarantee the invasion of measles in Italy.

We have no way to know how many infective individuals arrived with measles in Italy in 2017 but with the above assumptions and the method proposed here, it is possible, although very laborious, to backcalculate how many infected immigrants would be necessary to generate the observed 4,885 cases in 2007.

Table 1 shows the variables and parameters used in this section as well as their biological meanings.

### 2.2. Sensitivity Analysis

Since we are interested in the risk of disease introduction, only the first generation of cases is considered. In this section we calculate the sensitivity of the risk to the model's parameters. As the risk is dependent only on the “birth rate” of the infection, we calculate the sensitivity to the integral of this variable, that is, the number of cases. The incidence of the infection (“birth rate”) has been defined as *λ*(*t*) = *σI*_*H*_^*T*^(*t*)(*S*_*H*_(*t*)/*N*_*H*_). Therefore, the number of cases of measles, *Measles*_*cases*_, generated by the infected travellers is given by (2),

(8) $$\begin{array}{c} Measles_{cases}=\overset{\infty }{\underset{0}{\int }}\sigma I_{H}^{T}\left(\;t\;\right)\frac{S_{H}\left(t\right)}{N_{H}}dt \end{array}$$

where once again *I*_*H*_^*T*^(*t*) = *I*_*H*_^*T*^(0)exp⁡[−(*μ*_*H*_ + *γ*_*H*_ + *α*_*H*_)(*t* − *t*_0_)]*θ*(*t* − *t*_0_).

In order to estimate the sensitivity of the number of measles cases generated by the infected travellers, we simulated a SIR model, described in Appendix, with the parameters as in Table 1, and varied each one by 1 percent.

Table 2 shows the results of the sensitivity analysis according to the general (3). The results represent the relative variation in *Measles*_*cases*_, when we vary the parameters by 1%.

Therefore, the risk of measles invasion is less sensitive to the duration of the infection in the traveller, 1/*γ*_*H*_, than to the potentially infective rate *σ*. For every 1% variation in the *σ* parameter there will be a 1.01% of variation in the number of cases whereas for every 1% variation in the *γ*_*H*_ parameter there will be a 0.61% of variation in the average number of cases, in the first generation of the infection (that is, *t* = 1/(*μ*_*H*_ + *γ*_*H*_ + *α*_*H*_)). Note that the model is sensitive to neither the human mortality rate nor the additional mortality induced by the disease.

## 3. Case 2. Vector-Borne Infection

As in Case 1, we assume that a density of infected individuals arrives at *t* = *t*_0_. We wish to calculate the probability that these infected individuals produce zero latent mosquitoes, that is, *p*_*L*_*M*_=0_(*t*) for *t* > *t*_0_.

As in the previous section, we assume that the infected travellers infectiousness lasts on average 1/(*μ*_*H*_ + *γ*_*H*_ + *α*_*H*_) times units. Therefore,

(9) IHTt=IHT0exp−μH+γH+αHt−t0θt−t0

(note that (9) has the same form as (1)). However, the parameters *μ*_*H*_, *γ*_*H*_, and *α*_*H*_ are different and are defined in Tables 1 and 3. In Table 1 the values refer to measles whereas in Table 3 they refer to ZIKV.

We wish to estimate the probability that the infected traveller produces at least one latent mosquito along his/her infectiousness period. For this we used the same formulation as in Case 1 with the difference that in this case the “birth” term refers to the incidence of infection for mosquitoes, *λ*_*L*_*M*__(*t*). Note that, as in Case 1, we are interested only in the estimation of the risk of infection and, therefore, recovery from the infection is not considered in the risk estimation.

The incidence of the infection for the mosquitoes is given by

(10) $$\begin{array}{c} \lambda_{L_{M}}\left(\;t\;\right)=ac\frac{S_{M}\left(t\right)}{N_{H}}I_{H}^{T}\left(\;t-t_{0}\;\right)\theta \left(\;t-t_{0}\;\right) \end{array}$$

In (10) the number of susceptible mosquitoes *S*_*M*_(*t*) is obtained by solving

(11) dSMtdt=−acIHt+IHTtSMtNH+μMNM−SMt+dNMdt

In (11), the value of *N*_*M*_ and *dN*_*M*_/*dt* were obtained by the methods described in [24] considering a dengue outbreak. On the other hand, the value of *I*_*H*_(*t*) is obtained by solving the following system:

(12) dSMtdt=−acIHt+IHTtSMtNH+μMNM−SMt+dNMdtdLMtdt=acSMtIHt+IHTtNH−γMLMt−μMLMtdIMtdt=γMLMt−μMIMtdSHtdt=−abIMtSHtNH+μHNH−SHt+αHIHtdIHtdt=abIMtSHNH−μH+γH+αHIHtdRHtdt=γHIHt−μHRHtIHTt=IHT0exp−μH+γH+αHt−t0θt−t0NH=SHt+IHt+RHtSH0=NHIH0=RH0=0

Finally, in (12) *N*_*H*_ refers to the total local population density of humans.

Therefore, we can use (4a) and (4b) to calculate the *Risk*_*ZIKV*_(*n*, *t*) as

(13) RiskZIKVn,t=∑j=0minn,aaja+n−j−1a−1αta−j·βtn−j1−αt−βtj

and the risk (probability) of having no infected individuals is

(14) $$\begin{array}{c} Risk_{ZIKV}\left(\;0,t\;\right)=\alpha {\left(\;t\;\right)}^{a} \end{array}$$

In (13) and (14), as in Case 1, we assumed that the region to be studied has an area *A* and that the number of infected travellers arriving at *t* = *t*_0_ is *a* = *I*_*H*_(0)*A* (we set *a* = 1). In addition, *α*(*t*) = 1 − 1/(*e*^*ρ*(*t*)^ + ∫_0_^*t*^*λ*_*L*_*M*__(*τ*)*θ*(*τ* − *t*_0_)*e*^*ρ*(*τ*)^*dτ*), *β*(*t*) = 1 − *e*^*ρ*(*t*)^/(*e*^*ρ*(*t*)^ + ∫_0_^*t*^*λ*_*L*_*M*__(*τ*)*θ*(*τ* − *t*_0_)*e*^*ρ*(*τ*)^*dτ*), and *ρ*(*t*) = −∫_0_^*t*^*λ*_*L*_*M*__(*τ*)*θ*(*τ* − *t*_0_)*dτ*.

As in Case 1 the probability of at least one autochthonous case, after a suitable time, in a previously unaffected region can be calculated as follows:

(15) $$\begin{array}{c} P\left(\;\text{Number of secondary cases}>1\;\right)=1-\alpha^{a} \end{array}$$

The risk, therefore, is

(16a) $$\begin{array}{c} {Risk}_{ZIKV}=1-p_{L_{M}=0} \end{array}$$

where

(16b) $$\begin{array}{c} p_{L_{M}=0}=\alpha^{a} \end{array}$$

### 3.1. The Case of Zika Virus in Brazil

In this section we detail the calculation of the risk of Zika virus(ZIKV) invasion of an Aedes infested area where the virus has not been reported. The risk of invasion, *Risk*_*ZIKV*_(*t*), is defined as the probability of infected traveller will produce at least one secondary infection throughout the mosquitoes population along his/her infectiousness period.

The model's parameters, biological meaning, and values used in the simulations of this section are shown in Table 3.

We used this method to estimate the probability of ZIKV invading the city of Rio de Janeiro assuming the same mosquito density as in the dengue outbreak of 2012, the most important in the history of dengue in that city. Provided that the local Aedes population is at least as competent to transmit ZIKV as it is to transmit dengue, then the risk of invasion, as defined above, caused by an infected traveller arriving at the worst moment is around 22%. The 95% CI for the number of infected mosquitoes produced by one single infective traveller is (0.21-5.44). In other words, one infected mosquito will be produced with confidence interval between 0.21 and 5.44 mosquitoes per unit area.

We decided not to include diagrams for the two models (measles and Zika) to save editorial space, since the structure of both the Kermack-Mackendrick model [26] of the first case and the Ross-Macdonald model [18] for the second case are well known.

### 3.2. Sensitivity Analysis

In this section we calculate the sensitivity of the risk to the model's parameters. As in this case the risk is dependent only on the force of the infection, we calculate the sensitivity of the integral of this variable. The incidence of infection is defined as *λ*_*L*_*M*__(*t*) = *abI*_*M*_(*t*)(*S*_*H*_(*t*)/*N*_*H*_). Therefore, the number of cases of ZIKV, *ZIKV*_*cases*_, generated by the infected travellers, will be

(17) $$\begin{array}{c} ZIKV_{cases}=\overset{\infty }{\underset{0}{\int }}abI_{M}\left(\;t\;\right)\frac{S_{H}\left(t\right)}{N_{H}}dt \end{array}$$

where once again *I*_*H*_^*T*^(*t*) = *I*_*H*_^*T*^(0)exp⁡[−(*μ*_*H*_ + *γ*_*H*_ + *α*_*H*_)(*t* − *t*_0_)]*θ*(*t* − *t*_0_).

In order to estimate the sensitivity of the number of ZIKV cases generated by the infected travellers, we simulated a Ross-Macdonald model, described in Appendix, with the parameters as in Table 3, and varied each one by 1 percent.

Table 4 shows the results of the sensitivity analysis according to the general (3). The results represent the relative variation in *ZIKV*_*cases*_, when we vary the parameters by 1%.

Therefore, the risk of ZIKV invasion is orders of magnitude more sensitive to the biting rate and the probability of transmission from human to mosquitoes and vice versa, from mosquitoes to humans than the duration of the infection in the traveller, 1/*γ*_*H*_, or the mortality rate in humans *μ*_*H*_.

## 4. Discussion

In this note, we present two probabilistic models that intend to provide risks estimations of infectious diseases introduction into previously unaffected countries/regions. We analyse two distinct situations, one dealing with a directly transmitted infection and one dealing with a vector-borne infection. For calculating the risk in the first scenario we used a known model due to Bailey [25] (see also [27]) in which the disease spreads as a simple, nonhomogeneous birth process. The incidence of the infection is the main (in fact, the only) parameter in the model and this is justified by the fact that we are only interested in the risk of disease introduction rather than its full dynamics. This model allows the calculation, through a Probability Generating Function (3), that one single infective traveller arriving in a previously uninfected area produces one secondary, autochthonous case, along his/her infectiousness period (1). As a proof of principle, we apply this first model to the case of the current measles outbreak in Italy, triggered probably by one or more infective immigrants from non/undervaccinated areas of the world. This resulted in a risk, according to our definition, of 93%. Note that this is the probabilistic risk of one secondary case and as we estimate the basic reproduction number of measles in Italy in 2017 as equal to 5, it should be expected that one single infective traveller would introduce the infection in the region he/she arrives in. Even though the basic reproduction number, *R*_0_, is defined as the number of secondary infections one single infective individual will produce along his/her infectiousness period [26], in the determinist setting of an SIR model for measles in our example, the value of *R*_0_ is exactly 5. However, as the number of infective individuals in a given restricted geographical area tends to be small, it is important to calculate the chance that even when the conditions for the disease invasion to occur are present, none or just a small number of cases are produced and then vanishes. This is known as stochastic extinction. In the case of measles in Europe 2017, this chance is low (7.0%) even though we consider only a single infected traveller arriving in a very large area. As mentioned above, the expected number of infected travellers that seeded measles in Italy in 2017 estimated by (4a) resulted in only 7 infective travellers that would be necessary to guarantee that measles would invade Italy.

The second probabilistic model proposed in this paper is intended to provide a way to calculate the number of local mosquitoes that would be infected by one single infective traveller along his/her infectiousness period. Like in the first model, this second model assumes that one infected traveller remains infective by an average period of (*μ*_*H*_ + *γ*_*H*_ + *α*_*H*_) units of time, after which he/she would either die of natural causes or recover from the infection or die of the disease. The model is simplified by the assumption that we calculated the probability that the infective traveller infects at least one mosquito, which turns into a latent mosquito. Then we calculated the probability that the latent mosquito would survive through the extrinsic incubation period, 1/*γ*_*M*_. This mosquito, when biting the susceptible local inhabitants, *S*_*H*_(*t*), with a rate *a* and probability of infecting *b*, would generate at least one autochthonous case with the probability given of 22%. This should be compared with the risk of 93% of measles invasion because measles in more infective than Zika, as reflected by it bigger value of their reproductive numbers (5 versus 2, see [28]).

The essential difference between the two models presented in this paper is that the first, a nonhomogeneous simple birth process, is more suitable for directly transmitted infections, like the measles example showed, whereas the second, by including all the intermediate steps in the chain of transmission, is more suitable for indirectly transmitted infection, as exemplified by a vector-borne infection.

Finally, a note of caution with respect to the model's limitations is necessary. The main limitation of both models presented in this note is the assumption of homogeneously mixing population. According to this assumption, an infected traveller would have the same probability of interact with any susceptible individual of the local population. This is very unlikely to be the case in real outbreaks. Therefore, spatial heterogeneities should be included in the model to make the results more realistic, a task for future works on this line of research.

It should be clear that the results presented in this paper should not be taken as definitive and were presented only as a proof of concept and to illustrate the methods proposed. In order to complete the analysis of the risk of disease invasion by infective travellers of areas previously free of the infection it would be necessary to address the other component of the risk, namely the human movement from infected to noninfected areas by, for instance, the use of gravity-diffusion models [29].

## Figures and Tables

**Table 1:** Model variables and parameters, their biological meaning, and values for the measles case. The parameter values were taken from [18].

Parameter/Variable	Meaning	Value
*S*_*H*_(*t*)	Density^+^ of susceptible individuals	*S*_*H*_(0) = 20% of Italy Population
*I*_*H*_^*T*^(*t*)	Density of infected travelers	*I*_*H*_^*T*^(0) = 1
*I*_*H*_(*t*)	Density of infected individuals	*I*_*H*_(0) = 0
*N*_*H*_(*t*)	Density of individuals	Italy Population
*μ*_*H*_	Human natural mortality rate	1.10×10^−3^ month^−1^
*α*_*H*_	Additional Measles mortality rate	6.00×10^−5^ month^−1^
*γ*_*H*_	Measles recovery rate	3.33 month^−1^
*σ*	Potentially infective contact rate	10

^+^density means “number of individuals per unit area.”

**Table 2:** Sensitivity analysis. Relative variation in the number of cases when we vary the parameters by 1% per time unit in the first generation of the infection.

**Sensitivity of the number of cases to the parameters**	
**Parameter**	**Relative variation (**%**)**
*σ*	1.01
*μ*_*H*_	0.00
*γ*_*H*_	(-) 0.61
*α*_*H*_	0.00

**Table 3:** Model parameters, their biological meaning, and values for the ZIKV case. The parameter values were taken from [18].

Parameter	Meaning	Value
*a*	*A. aegypti* biting rate	10.040 month^−1^
*b*	Fraction of bites actually infective to humans	0.610
*μ*_*H*_	Human natural mortality rate	0.001 month^−1^
*α*_*H*_	ZIKV mortality rate	negligible
*γ*_*H*_	ZIKV recovery rate	0.080 month^−1^
*γ*_*M*_	Latency rate in mosquitoes for ZIKV	0.320 month^−1^
*μ*_*M*_	Natural mortality rate of mosquitoes	2.330 month^−1^
*c*	*A. aegypti* susceptibility to ZIKV	0.530

**Table 4:** Sensitivity analysis. Relative variation in the number of ZIKV cases when we vary the parameters by 1% per time unit.

**Sensitivity of the number of ZIKV cases to the parameters**	
**Parameter**	**Relative variation (**%**)**
*a*	1.48
*c*	7.4 × 10^−1^
*b*	7.4 × 10^−1^
*μ*_*H*_	0.0
*γ*_*H*_	(-) 1.5 × 10^−3^

## Data Availability

Data used are described in a table in the manuscript.

## Appendix

### A.

In this Appendix we describe the SIR [26] model used for the sensitivity analysis of Case 1.

The model consists of 4 variables, susceptible individuals, denoted *S*_*H*_(*t*), infected individuals, denoted *I*_*H*_(*t*), infected travellers, denoted *I*_*H*_^*T*^(*t*), and removed, denoted *R*(*t*). The model is described by the following system:

(A.1) dSHtdt=−σIHt+IHTtSHtNH−μHSHt+μHIHt+RHt+IHTt+αHIHt+IHTtdIHtdt=σIHt+IHTtSHtNH−μH+γH+αHIHtdIHTtdt=−μH+γH+αHIHTtdRHtdt=γHIHt+IHTt−μHRHtNHt=SHt+IHt+IHTt+RHt

where the parameters were described in Table 1 of the main text.

We then calculated the total number of cases produced by one infected traveller, according to

(A.2) $$\begin{array}{c} Measles_{cases}=\overset{\infty }{\underset{0}{\int }}\sigma I_{H}^{T}\left(\;t\;\right)\frac{S_{H}\left(t\right)}{N_{H}}dt \end{array}$$

which is (2) of the main text.

Finally, we varied each parameter by 1% of theirs baseline values and calculated the relative variation in the number of cases.

### B.

In this Appendix we describe the Ross-Macdonald [18] model used for the sensitivity analysis of Case 2.

The populations involved in the transmission are human hosts and mosquitoes. Therefore, the population densities for humans are divided into the following compartments: susceptible individuals, denoted *S*_*H*_(*t*), infected individuals, denoted *I*_*H*_(*t*), infected travellers, denoted *I*_*H*_^*T*^(*t*), and removed, denoted *R*(*t*). The mosquitoes are divided into susceptible mosquitoes, *S*_*M*_(*t*), infected and latent mosquitoes, *L*_*M*_(*t*), and infected and infectious mosquitoes, *I*_*M*_(*t*). The parameters appearing in the model are defined in Table 3.

The model is defined by the following:

(B.1) dSHtdt=−abIMtSHtNH+μHNH−SHt+αHIHtdIHtdt=abIMtSHNH−μH+γH+αHIHtdIHTtdt=−μH+γH+αHIHTtdRHtdt=γHIHt+IHTt−μHRHtdSMtdt=−acSMtIHt+IHTtNH+μMLMt+IMtdLMtdt=acSMtIHtNH−γMLMt−μMLMtdLMTtdt=acSMtIHTtNH−γMLMt−μMLMtdIMtdt=γMLMt−μMIMtdIMTtdt=γMLMTt−μMIMtNH=SH+IH+RHNM=SM+LM+LMTt+IM+IMTt

We then calculated the total number of cases produced by one infected traveller, according to

(B.2) $$\begin{array}{c} ZIKV_{cases}=\overset{\infty }{\underset{0}{\int }}abI_{M}\left(\;t\;\right)\frac{S_{H}\left(t\right)}{N_{H}}dt \end{array}$$

which is (17) of the main text.

Finally, we varied each parameter by 1% of theirs baseline values and calculated the relative variation in the number of cases.
